# Cases of Amaurosis Treated by Strychnine

**Published:** 1830-10-01

**Authors:** 


### CLINICAL REVIEW.

### VI.

### Some Cases of Amaurosis treated by Strychnine.

[Reported, by Dr. Heathcote, from the Royal Infirmary of Edinburgh.]

In September, 1826, M. Lembert read his " Essai sur le Methode Endermique" before the Royal Academy at Paris. This essay consists of experiments with various medicines applied externally upon blistered surfaces, or by injection into the cellular substances. Amongst others, the vegetable alkali called strychnia (being prepared from the strychnos nux vomica) was successfully employed in a case of hemiplegia following apoplexy, and its more violent and injurious effects restrained by the same application of the narcotic principle of opium.

From this Essay, I believe, Dr. Short conceived the idea of applying strychnia for the cure of amaurosis, and that conception has been attended with the most gratifying result; for, although few only have benefited, yet what success can a physician find more gratifying than to have restored sight to one blind, and opened the way for others to remove so calamitous an affliction? Having stated the occasion which led to the use of strychnia in these cases, I have only to show its mode of preparation, and the manner in which it has been applied, before I proceed to the cases themselves.

The powder of the nux vomica is first boiled in water, and the decoction evaporated until it acquires the consistence of syrup; lime is then added, which, uniting with the acid, liberates the strychnia, which may then be separated by means of alcohol, in which it is very soluble, and may be obtained by crystallization.

To apply it:—a small blister, about the size of a crown-piece, is first put upon the temple or forehead, and upon which, having risen, and the cuticle being removed, $\frac{1}{4}$ of a grain of strychnia, finely levigated, is dusted, and a piece of simple dressing is placed.

I now proceed to detail those cases in which this remedy, so applied, has been attended with any benefit. But, with regard to the majority which have not been benefited, in order to save the time of my readers, I have arranged their cases in a table, which embraces all the particulars of interest; their similarities and differences, in as short a compass as possible.

*Case* 1. Peter Hamilton, æt. 22, an iron founder, admitted June 16th, 1829. Can only distinguish light from darkness. Both pupils are much dilated, the right more than the left. The iris in both is sensible to the stimulus of light. The eyes are clear, and, with the exception of a slight squint, present a natural appearance. This state of vision has continued nearly the same for two years. His account of its commencement is as follows.

Having been for some years daily working under exposure to the heat and light of an iron-founder's furnace, he became affected with indistinctness of vision, accompanied by flashes of light when looking at minute objects, or when stooping. This indistinctness became gradually more and more obscure for 15 months. At the end of this time he could only distinguish light from darkness, and has remained in that state nine months. His general health had all along been quite good.

17th, (1st day). The temples were shaved, and $\frac{1}{8}$ of a grain of Strychnia, dusted the next day on each side.

The next report I find, was made six days after this, and is as follows.

23rd June (6th day). Within the last week a blister has been twice in succession applied to each temple, and to the raw surfaces, first $\frac{1}{8}$, then $\frac{1}{4}$, and to-day, $\frac{1}{2}$ a grain of the powder of strychnia.

This improvement has been progressive from the state of distinguishing only between light and darkness to the perception of the particular finger or number held up between him and the window. The pupils are less dilated and the iris readily contractile—strabismus is almost gone—tongue rather foul—bowels open.

24th. (7th day) Blisters were renewed.

25th. (8th day) Can to-day distinguish colours pretty readily, especially with left eye; the iris of which is less sensible than that of the right—$\frac{1}{2}$ a grain was applied to each temple.

26th. (9th day) Still continues to improve, and can distinguish yellow and red colours—some headache, and tongue much loaded, and white—$\frac{3}{4}$ of a grain to each surface, a cathartic mixture.

27th. (10th day) Less headache—sight considerably improved, for he can distinguish print from writing—one grain applied to each surface.

28th. (11th day) Vision more distinct. Had an additional grain and $\frac{1}{4}$ yesterday. Had $1\frac{1}{2}$ on each surface.

29th. (12th day) Blistered surfaces have healed.

July 2nd. (15th day) Had $1\frac{3}{4}$ grains on the 30th. On the 1st an attack of rigors, debility, sickness, vertigo, and head ache, which are now gone, but feels weak. Can now clearly distinguish objects placed at the distance of some paces, and reads easily the hour upon a watch, by evening twilight—iris of both eyes quite sensible. Intermit strychnine.

4th. (17th day) Sight still further improved. Renew the blisters and $\frac{1}{4}$ grain of the powder.

13th. (26th day) Can now distinguish objects clearly at considerable distances. Pupils continue more contracted, although less than naturally. Strychnia from $\frac{1}{4}$ to $\frac{3}{4}$ of a grain, has been applied as before.

26th. (39th day) Strychnia has not been applied since last report, from a sensation of violent heat over the skin.

Aug. 4th. (48th day) Since the last report, two grains have been applied to each temple, without any obvious effect. But improvement in vision continues.

16th. (56th day) Has had two grains on each temple for eight days. Repeat blisters, and apply $2\frac{1}{2}$ grains to each surface.

Sept. 8. (79th day) Since the last report he has been applying the strychnia every day, from 2 to $3\frac{1}{2}$ grains on each temple without any constitutional effect, but with continued improvement in his sight. There were some days of intermission when the blisters were obliged to be removed. Yesterday left the infirmary, and attempted to work, but found that the act of stooping occasioned dimness of sight, and he returned the next day, and resumed the strychnine to the extent of three grains on each temple, and continued ijss to the 13th. It was then altogether omitted, and on the 31st, the report is, that he could see perfectly, and he was ordered to apply the vapour of ammonia for a few days; the eyes appeared quite natural, the squinting gone, and he was enabled to tell the time upon the iron church clock, from the infirmary windows.

*Case* 2. Andrew Drummond, æt. 34, a ship carpenter, admitted Oct. 2nd, 1829. This patient can only distinguish light from darkness, a window from the wall. The pupils of both eyes are rather contracted, and there is but little sensibility of the iris. In the left eye there is a ring of opaque substance, the remains of a cataract, the centre of which was removed by an operation: but although the light was thereby admitted, he was still unable to see. This ring can only be observed when the pupil is dilated. This state of vision, with little change, has continued six years. His account of its commencement is as follows.

He had been employed as a flax dresser, six months before he was attacked with fever at Dundee. This was about six years ago. But some weeks previous to this attack, dimness of sight had gradually come on, and then, during the delirium of fever, his sight was totally lost, and likewise his hearing.

During his convalescence he regained his hearing, but remained nearly quite blind. Sometimes, however, distinguishing light from darkness. A year after this he was admitted into the infirmary, had the centre of the cataract removed from the left eye, was blistered, and had setons, both in the nucha and in the inside of the arms. But he left the hospital without receiving any benefit to his sight. Two years since he returned to the house, underwent medical treatment, took mercury, renewed his blisters and setons, and left the house again, without any distinct improvement, but only thinks he saw a little more clearly. He has since his fever enjoyed good general health.

Oct. 3rd. (1st day) A blister having been applied to the right temple, yesterday $\frac{1}{4}$ of a grain of strychnia was dusted upon the raw surface, the cuticle being removed, and states, that he saw a little with the right eye, about six hours after the application, and that almost immediately after, he felt a shooting pain across his forehead, but no other sensation—one grain to be applied.

5th. (3rd day) Says he did not feel the application of the whole grain so much as the first $\frac{1}{4}$, but thinks his sight still improved—full diet—blisters renewed.

7th. (5th day) One grain since last report. Can now distinguish large from small letters—some vertigo last night; $1\frac{1}{4}$ grain, and aqua ammoniæ.

10th. (8th day) Since last report a fresh blister, and three grains have been applied as before. Sees the iron church steeple from the window, and distinguishes the colours of the ward, which he could not do yesterday—one grain as before, and a cathartic mixture.

13th. (11th day) Since the 8th, $2\frac{1}{2}$ grains have been applied; noticed yesterday, with his left eye, the bars of the window—two blisters have been since applied, and have risen well; strychnia omitted yesterday, and no further improvement in sight—apply 1½.

18th. (16th day) Five grains since the 13th, and a renewal of the blisters—complains of the light affecting his eyes, which he had not experienced before.

22nd. (20th day) Three grains since the 18th, with alternate blisters on each temple. Can to-day see men walking at a distance, which he could not do yesterday.

Nov. 7th. Since 22nd, 19½ grains have been applied, at most two grains and ½ at once. His sight gradually improving, and the intolerance of light removed entirely. Strychnia has been intermitted, and his appetite improved by the tincture of calumba. He can now see (11th Nov.) to read the hour upon a common watch—but he cannot yet distinguish small print.

*Case* 3. William Smith, æt. 31, a mason, admitted October 13th, 1829. The pupils are not much dilated, and the iris, though sluggish, is sensible: over the left cornea there is a slight muddiness. The eyes are in other respects natural. He can only distinguish light from darkness, but the left eye is more dark than the right. This state of vision has continued, with one period of improvement, about 12 years. His account of its commencement is as follows:

Twelve years ago, while going to his work, as a mason, he perceived a dimness in his sight, as if he were looking through smoke (according to his own description). In three days from this time he became quite blind; and cannot assign any cause for it, his general health having been quite good. Blisters were kept open, on his temple, without effect. About a year afterwards, in the Autumn, his sight partially returned, so that he was enabled to shear, by strong day light, but was obliged to be led home in the evening. Since which time he has been blind as described, and used no remedy.

16th. (2nd day) Quarter of a grain of strychnia was applied yesterday to the raw surface of each blistered temple. No change in sight—sensation of metallic taste an hour after the application of the powder—had pain also in the balls of the eyes—bowels regular.

22nd. (8th day) Since the last report, has applied 8 grains—1½ daily, and a blister alternately to each temple when necessary to keep the surface raw. Had slight headache yesterday, and a pricking sensation in his arms and legs.

28th. (14th day) Since the last report, has applied with increase or diminution daily, according to the constitutional effects 8¾. Forehead to-day is swollen, and the pricking sensation is felt to the finger's ends—intermit strychnia. Thinks that both eyes are slightly improved, but nothing more definite can be stated.

Nov. 1st. (18th day) The swelling of the forehead being removed by the intermission of the powder for two days and the bowels opened by cathartic mixture—he resumed its application on the 30th, beginning with ½ a grain, the next day ¾. Can now see the bars of the window, and observes the book before him, which he has never done before—says that his eyes feel stronger.

5th. (22nd day) Went out into the street, and could perceive people approaching him, which before he was unable to do. Has had since the last report, 3½ grains.

10th. (27th day) Since the last report, 2¼ grains have been applied without any obvious general effects. Can now see below him for the first time, and distinguishes articles placed upon the table, and is able to direct his hand to them—nebulous appearance of the left cornea continues—strychnine intermitted, there being none in the infirmary.

Since these cases were read before the Physical Society of Edinburgh, a few instances of the success of strychnine in amaurosis have, I believe, been published; and some remarks have been elsewhere made upon the efficacy of the remedy. But these related facts and remarks do not appear to me to have been embodied in sufficient force to arrest the attention of medical practitioners; and there remains no prominent exhibition of amau-

### CASES OF AMAUROSIS TREATED WITH STRYCHNINE.—*Royal Infirmary, Edinburgh, Oct. 1829.*

| NAMES. | Age. | Trade. | Time Blind. | State of Eyes. Right. | Left. | Pupils. | Iris. | Eye-ball. | Causes and Mode. | Remedies. | Changes. | Quantity of Strychnine and its Effects. |
|---|---|---|---|---|---|---|---|---|---|---|---|---|
| John Sinclair, | 20 | —— | Infancy | Sees lt. from d. | Quite blind | Much dilated | Contractile | Trembling | Born, | —— | None | 5 grains and 1 qr. without any effect, in 5 days. D. |
| John Kennedy, | 18 | None | 15 years | Cataract | Sees to guide | Natural | Contractile | —— | Measles. | Medicines. | None | 6 grains in 5 days without any effect. D. |
| And. Henderson, | 26 | Weaver | 6 years | Quite blind | Quite blind | Much dilated | Insensible | Trembling | Headache & oph. 6 months going | Bled—blistered | None | 7 grains in 5 days without any effect. D. |
| John Pringle, | 17 | Weaver | 6 years | Sees light from darkness | Sees to guide | Contracted | & Contractile | —— | Head-ache, sight failed in 4 months | Blisters and Collyria | None | 3 grains in 3 days caused giddiness and tremors—3 more—no effect. Œdema of eyelids. D. |
| John M'Knee, | 24 | —— | 20 years | Do. do. | Quite blind | Contracted | Insensible | —— | None. Gradual dimness | 4 cat. removed from each eye | Slight, but relapsed | 2 and half grains in 5 days, (one omitted). No effect. D. |
| George Gall, | 16 | —— | 6 years | Sees to guide | Quite blind | Contracted | Sluggish | —— | Head-ache from blow | —— | None | 2 & half grs. in 5 days (one omit.) head-ache—took 3 grains more —iris more sensible—vision improved—7 grains more in 7 days —no effect. Int. |
| John Gibson, | 32 | —— | 22 years | Light from dark | Light from dark | Much dilated | Insensible | —— | Gradual dimness 9 to 12 months. | Blist.—mercury, electricity | No change | 4 and half in 4 days—no effect. Do. unrelieved. |
| Alex. Millar,* | 15 | —— | 5 years | Quite | blind | Dilated | Contracted | Tremulous | Hydrocephalus grad. in 7 months | Bled, blistered repeatedly | None* | Half a grain 1st day—head-ache —5 grs. more in 4 days—*hearing* but no sight—5 grs. more in 4 days, no effect—1-qr. more behind the ears—no further effect. —Omit. |
| William Grieve, | 45 | Groom | —— | Obscure | Opake | Natural | Contractile | —— | Left eye sudden, right cataract. | Cat. removed, various | None | 7 1-qr. grs. in 7 days without any effect. Do. by desire. |
| Thomas Lamb, | 44 | —— | 21 years | Can just | guide himself. | Natural | Contractile | —— | Fever—blind during delirium | Slight improve | ment | Half-grain caused pricking, vertigo, debility, headache—1-qr. more, after 5 days—return of headache and faintness. D. |
| Peter Hamilton, | 22 | Iron-founder | 2 years | Light from | darkness | Much dilated | Sluggish | Squint | Gradual dimness | Collyria | None | About 2 grs. in 6 days, progressive in sight—9th day, after 1 grain more, head-ache—15th day, rigors, sickness, vertigo, tell the hour by the watch. |
| And. Drummond, | 34 | Carpenter | 6 years | Ditto | Do. and ring of cataract | Contracted | Sluggish | —— | Dimness with loss in fever | Blisters—setons merc. and med. | No permanent | 1-qr. grs. after 6 hours gave some sight, with headache across forehead—2 and qr. grs. more—vertigo, great improvement in sight |
| William Smith. | 31 | Mason. | 12 years | Ditto | Do. cloudy | Natural | Sluggish | —— | Dimness in three days lost. | Open blisters | One suture slight imp. | 1-qr. grains—head-ache, pains in eye-balls. After 18 gr. more, & frequent constitutional sympt. much improvement in sight. |

rosis, treated and cured by strychnine, to which medical men may refer, to encourage the attempt in future cases, and to warrant the probability of success.

I think the foregoing cases will supply this desideratum; and when it is known that two additional instances of success, one before and one since the date of these reports, have followed the same treatment, we are justified in encouraging and continuing this mode of cure.

It should be remembered that the use of this remedy proved injurious in no case where it was unattended with success.

A slight degree of sickness, giddiness and head-ache, with twitching of the limbs, were the general symptoms complained of. In one case erysipelas of the face occurred, which immediately subsided upon the omission of the strychnine and the use of opium, which is its proper antidote. It is remarkable, too, that in most of those cases where this remedy proved ineffectual, very slight or no constitutional symptoms were produced. One abandoned it through fear—others from impatience. It will be seen, also, by a reference to the table, that the deafness, though not the sight, was alleviated in one instance by its application. All three cases, with one exception, were taken from the Blind Asylum in Edinburgh.

There is one more circumstance attending the use of strychnine in this manner which it is necessary to observe, and that is, that there are individual constitutions which exhibit very opposite symptoms to those generally produced by this medicine. A gentleman at Bristol, whom I attended, had been afflicted with amaurosis, combined with cataract, and had been blind for two years. This gentleman had applied to his temples about seven grains of strychnine in the usual way before any effects manifested themselves; and then, instead of the usual exciting effect, he was seized with numbness and immobility of the lower extremities, which gave way to a few doses of opium and aperient medicines.

*Note by the Editor.*

In the Medical Gazette, five cases are reported from the Westminster Ophthalmic Infirmary, treated on the above plan. In one case out of the five, where the amaurosis had lasted nearly two years, "evident and considerable benefit" ensued. In the other four cases no improvement resulted. Still, in a disease so nearly hopeless as amaurosis, the prospect held out by Dr. Heathcote's and Mr. Guthrie's experiments is not to be contemned.

